# Development and Optimization of a GMP-Compliant Manufacturing Process for a Personalized Tumor Lysate Dendritic Cell Vaccine

**DOI:** 10.3390/vaccines8010025

**Published:** 2020-01-14

**Authors:** Caroline Boudousquié, Valérie Boand, Emilie Lingre, Laeticia Dutoit, Klara Balint, Maxime Danilo, Alexandre Harari, Philippe O. Gannon, Lana E. Kandalaft

**Affiliations:** 1 Department of Oncology, Centre Hospitalier Universitaire Vaudois, 1011 Lausanne, Switzerland; valerie.boand@gmail.com (V.B.); Emilie.Lingre@chuv.ch (E.L.); Laeticia.Dutoit@chuv.ch (L.D.); Klara.Balint@chuv.ch (K.B.); Alexandre.Harari@chuv.ch (A.H.); Philippe.Gannon@chuv.ch (P.O.G.); 2 Department of Oncology, Ludwig Institute for Cancer Research, University of Lausanne, 1011 Lausanne, Switzerland; Maxime.Danilo@chuv.ch

**Keywords:** immunotherapy, dendritic cell, vaccine, manufacturing, GMP, tumor lysate

## Abstract

With the emergence of immune checkpoint inhibitors and adoptive T-cell therapies, there is a considerable interest in using personalized autologous dendritic cell (DC) vaccines in combination with T cell-targeting immunotherapies to potentially maximize the therapeutic impact of DC vaccines. Here, we describe the development and optimization of a Good Manufacturing Practice (GMP)-compliant manufacturing process based on tumor lysate as a tumor antigen source for the production of an oxidized tumor cell lysate loaded DC (OC-DC) vaccine. The manufacturing process required one day for lysate preparation and six days for OC-DC vaccine production. Tumor lysate production was standardized based on an optimal tumor digestion protocol and the immunogenicity was improved through oxidation using hypochloric acid prior to freeze-thaw cycles resulting in the oxidized tumor cell lysate (OC-L). Next, monocytes were selected using the CliniMACS prodigy closed system and were placed in culture in cell factories in the presence of IL-4 and GM-CSF. Immature DCs were loaded with OC-L and matured using MPLA-IFNγ. After assessing the functionality of the OC-DC cells (IL12p70 secretion and COSTIM assay), the OC-DC vaccine was cryopreserved in multiple doses for single use. Finally, the stability of the formulated doses was tested and validated. We believe this GMP-compliant DC vaccine manufacturing process will facilitate access of patients to personalized DC vaccines, and allow for multi-center clinical trials.

## 1. Introduction

Often referred to as the most potent antigen presenting cells (APC), dendritic cells (DC) have been used since the mid-1990s in clinical trials for therapeutic vaccination of cancer patients [1]. The proven immunogenicity of DC-based vaccines and their safety profile were very promising, but objective clinical responses were not as positive as initially expected [2,3]. Over the past decade, the emergence of immune checkpoint inhibitors and adoptive T-cell therapy in the immunotherapy landscape reinvigorated the interest in DC-based therapies. Indeed, the use of personalized autologous DC vaccines in combination with T-cell-targeting immunotherapies appears as an appealing opportunity for vaccines to reach their full therapeutic potential. To this date, one of the major challenges for DC vaccination remains a cost-effective GMP-compliant process standardized in the EU, in Switzerland, and in the United States.

Additionally, the choice of the antigen(s) to be loaded onto DCs is critical for vaccine efficiency. The best strategy for selecting antigens appears to be the use of several antigens, and, preferably, neo-antigens resulting from the patient’s own tumor’s mutational load [4,5,6]. Indeed, as neo-antigens are not expressed by healthy tissues, they can be recognized as non-self by the immune system, and are thus a target of choice for immunotherapy. To avoid the long and expensive process of neo-antigens identification and production [7,8,9,10], tumor lysate represents an attractive source of antigens for indications where surgery can be performed.

Tumor lysate is a cheap and safe source of antigens containing the full repertoire of the patient’s specific tumor associated antigens (TAA), including neo-antigens. A known disadvantage of the use of tumor lysate is its limited immunogenicity. However, several approaches were elaborated to overcome this limitation. A recent study from our group demonstrated a higher immunogenicity using a tumor lysate oxidized with hypochloric acid [11,12]. This study was translated into a phase I clinical trial [13] where ovarian cancer patients were treated with autologous oxidized lysate loaded-DC in combination with cyclophosphamide and bevacizumab. An enhanced immune response associated with a longer progression-free survival (PFS) was observed. These promising results encouraged us to transfer the OC-DC manufacturing process to the GMP Cellular Manufacturing Facility of the Department of Oncology of the Centre Hospitalier Universitaire Vaudois (CHUV) to optimize it and adapt it to current requirements for GMP vaccine production in Switzerland.

In this paper, we present the results from the process development work performed to optimize this GMP-compliant OC-DC vaccine production process. Oxidized tumor-lysate production was adapted to the use of GMP-compliant reagents with the tumor digestion optimized by comparison of passive (rotating wheel) and active (GentleMACS dissociator, Miltenyi, Bergisch Gladbach, Germany) dissociation. Monocytes isolation using the CliniMACS Prodigy system was tested and purified monocytes were successfully differentiated into immature dendritic cells (iDC) after five days of culture in the presence of IL-4 and GM-CSF. iDC were loaded with OC-L on day 5 and maturated on day 6 with a GMP-compliant maturation cocktail (MPLA/IFNγ). Cells were harvested 6 to 8 h after the addition of the maturation cocktail, and cryopreserved as OC-DC vaccine doses (5–10 × 10^6^ cells per dose). The OC-DC cells phenotype was assessed by flow cytometry and functionality was determined by COSTIM assay and IL12p70 production evaluation by Enzyme Linked ImmunoSorbent Assay (ELISA) on the final batch prior to cryopreservation. Moreover, we evaluated and optimized the OC-DC vaccine dose reconstitution (thawing, washing, and formulation in syringes). Finally, the process was integrated for manufacturing within a Grade D clean room environment including the validation of a quality control strategy suitable for Phase I clinical trial.

This production process proved to be efficient and highly reproducible, thereby confirming its suitability as a reference process for DC vaccine GMP manufacturing in a Grade D clean room using a Grade A isolator for all open steps.

## 2. Materials and Methods

### 2.1. Samples Procurement and Transport

All subjects gave their informed consent for inclusion before they participated in the study. The study was conducted in accordance with the Declaration of Helsinki, and the protocol was approved by the Ethics Committee of Canton Vaud, Switzerland (CER-VD 426/15, 2015).

The collection and transportation of tumor samples and leukapheresis bag were performed according to local standardized operating procedures.

### 2.2. Oxidized Whole Tumor Cell Lysate (OC-L) Preparation

The fresh tumor material was received in transport media composed of Roswell Park Memorial Institute medium (RPMI) supplemented with 1% Penicillin Streptomycin (Gibco by Life Technologies, Grand Island, NY, USA and Amimed by Bioconcept Allschwil, Switzerland, respectively) and manually processed into small fragments (approximately 1–3 mm^3^). The tumor fragments were used fresh or following cryopreservation into 90% Human serum (Biowest, Nuaillé, France) and 10% DMSO (Miltenyi Biotec, Bergisch Gladbach, Germany). The dissociation was performed using RPMI 1640 supplemented with DNase I at 30 IU/mL (Roche, Boulogne-Billancourt, France) and collagenase at 0.3 PZ units/mL (Nordmark, Uetersen, Germany) followed by an incubation either overnight at RT on a rotating mixer or on the GentleMACS (Miltenyi Biotec) using a tumor dissociation of three cycles of 30 min at 37 °C. The lysate was then centrifuged, washed in Dulbecco’s phosphate-buffered saline (DPBS) (Gibco by Life Technologies, Grand Island, NY, USA) and a cell count was performed. For the oxidation step, the lysate was resuspended at 10 × 10^6^ cell/mL in DPBS containing 60 µM HOCl (Amukina Med from ACRAF, Casella, Italy) and incubated for 1 h at 37 °C. After oxidation, two washes in DPBS were performed, cells were resuspended at 1 × 10^6^ cell/mL in cell culture media, and exposed to a minimum of five (5) freeze-thaw cycles. At the end of the procedure, no viable cell must be detected using Trypan blue exclusion method. The OC-L samples were stored at −80 °C until further use.

The presence of HOCL in OC-L was measured using a colorimetric hypochlorite detection kit (Abcam, cat. N° ab219929, Cambridge, UK) and following manufacturer’s instructions.

### 2.3. Generation of Monocyte-Derived Dendritic Cells

Monocytes were purified from fresh leukapheresis using positive selection of CD14^+^ cells on the CliniMACS Prodigy (Miltenyi Biotec). After purification, monocytes were counted and resuspended at 1 × 10^6^ cell/mL in CellGro GMP DC medium (CellGenix, Freiburg, Germany) complemented with 2% human serum, IL-4 (250 IU/mL, CellGenix) and GM-CSF (500 IU/mL, CellGenix) before transferring into cell factory flasks. Monocytes were incubated for 5 to 6 days at 37 °C, 5% CO_2_. Part of immature DC (iDC) were harvested at day 5 or 6 using cold CliniMACS buffer (Miltenyi Biotec) and cryopreserved either in 90% Human serum, 10% DMSO or in CS10 (BioLife Solutions, Bothell, WA, USA). On day 5 or 6, the remaining iDC were loaded with OC-L for a maximum of 16 h at a cell number ratio of OC-L:iDC 0.5-1:1. On day 6 or 7, the OC-L loaded iDC were matured for 6 to 8 h in the presence of IFNγ (2000 IU/mL, Boehringer Ingelheim, Basel, Switzerland) either with lipopolysaccharide (LPS, 60 EU/mL, Sigma, Darmstadt, Germany) or Monophosphoryl Lipid A (MPLA, 0.6 µg/mL, Avanti Polar Lipid, Alabaster, AL, USA). The matured cells were finally harvested using cold CliniMACS buffer and the OC-DC doses were cryopreserved at 5–10 × 10^6^ cell/mL in either 90% human serum, 10% DMSO, or in CS10 using CoolCell boxes (Biocision by Brooks life science systems, Chelmsford, MA, USA).

### 2.4. Formulation of OC-DC Vaccine Doses

Cryopreserved vaccine doses were thawed using a thawing solution of RPMI complemented with 1% albumin (Octapharma, Lachen, Switzerland), centrifuged at RT and resuspended in NaCl 0.9% (B. Braun) before being drawn into two 1-mL syringes.

### 2.5. Phenotypic Analysis by Flow Cytometry

The cells from leukapheresis, the monocytes and the DC harvested at different culture time were stained with CD14 PeCy7, CD86 APC, and HLA-DR PE (all antibodies from Biolegend, San Diego, CA, USA). Dead cells were excluded using the LIVE/DEAD™ Fixable Near-IR viability dye (Invitrogen, Carlsbad, CA, USA). Briefly, cells were washed and resuspended in FACS buffer (PBS (Bichsel, Interlakeun, Switzerland), 2% FBS (Gibco by Life Technologies, Grand Island, NY, USA), 2 mM EDTA (Life Technologies, Grand Island, NY, USA)) prior to incubation with Fc block reagent (Miltenyi Biotec, Bergisch Gladbach, Germany) for 5 min at 4 °C. The antibody mix was added before an additional 15 min incubation at 4 °C and a final cell wash in FACS buffer. Samples were acquired on a MACSQuant (Miltenyi Biotec, Bergisch Gladbach, Germany) and results were analyzed using MACSQuantify (version 2.1, Miltenyi Biotec, Bergisch Gladbach, Germany) or FlowJo (version 10.2, BD Biosciences, San Jose, CA, USA).

### 2.6. COSTIM Assay

Autologous T cells were purified from CD14 negative cells using magnetic selection (Pan T cell isolation kit, Miltenyi Bergisch Gladbach, Germany) and labelled with CFSE (CellTrace CFSE Proliferation Kit, Invitrogen, Carlsbad, CA, USA) following manufacturer’s instructions. T cells were counted and resuspended at 1 × 10^6^ cells/mL in AIM-V medium (Gibco by Life Technologies, Grand Island, NY, USA). Monocytes, iDC and OC-DC were thawed, washed and resuspended at 1 × 10^5^ cells/mL in AIM-V medium. T cells were co-cultured with monocytes, iDC or mDC in a 96-well plate in the presence or absence of anti-CD28 (1 µg/mL, BD Biosciences) and/or anti-CD3 (0.005 µg/mL, Miltenyi Biotec Bergisch Gladbach, Germany). Cells were incubated at 37 °C, 5% CO_2_ for 3 days, harvested and stained using CD4 APC-A750 (Beckman Coulter, Brea, CA, USA), CD8 PerCP Cy5.5 (Biolegend, San Diego, CA, USA) and DAPI (Invitrogen). Samples were acquired on a MACSQuant flow cytometer (Miltenyi Biotec, Bergisch Gladbach, Germany)) and analyzed using either MACSQuantify (Miltenyi, Bergisch Gladbach, Germany) or FlowJo (version 10.2, BD Biosciences, San Jose, CA, USA).

### 2.7. IL12p70 ELISA

Production of IL12p70 by DC was determined using enzyme-linked immunosorbent assay (ELISA). Culture supernatants were collected and stored at −80 °C before performing the ELISA analysis (Human IL-12 (p70) ELISA Kit, BD Biosciences, San Jose, CA, USA) following manufacturer’s instructions. The absorbance was determined using a microplate spectrophotometer (EPOCH2, BioTek, Sursee, Switzerland) with a measure at 450 nm and a wavelength correction at 570 nm.

### 2.8. Statistical Analysis

Statistical analysis included descriptive statistics using Mann–Whitney, Kruskall–Wallis or T test as appropriate. Statistical analysis was performed using GraphPad Prism (version 7.03, GraphPad software, San Diego, CA, USA).

## 3. Results

### 3.1. Oxidized Tumor Lysate (OC-L) Production Optimization

The OC-L manufacturing process was adapted from the method described by Chiang et al. [12] in order to render it GMP-compliant for Swiss regulatory requirements. During the process development steps, twenty-eight (28) OC-L batches were produced from either fresh or cryopreserved tumors. OC-L batches were prepared from ovarian and pancreatic tumor samples as these two indications are part of upcoming personalized DC vaccines clinical trial protocols.

A first step was to adapt the sourcing of reagents and consumables to meet GMP compliance in Switzerland. For the tumor dissociation steps, this involved changing the source of collagenase, DNase and HOCl for GMP grade reagents as opposed to what have been previously published [11]. For the oxidation of the tumor lysate, we used a medicinal solution of HOCl (Amukina Med from ACRAF) instead of a research grade reagent as required by the Swiss authorities.

**Figure 1:**
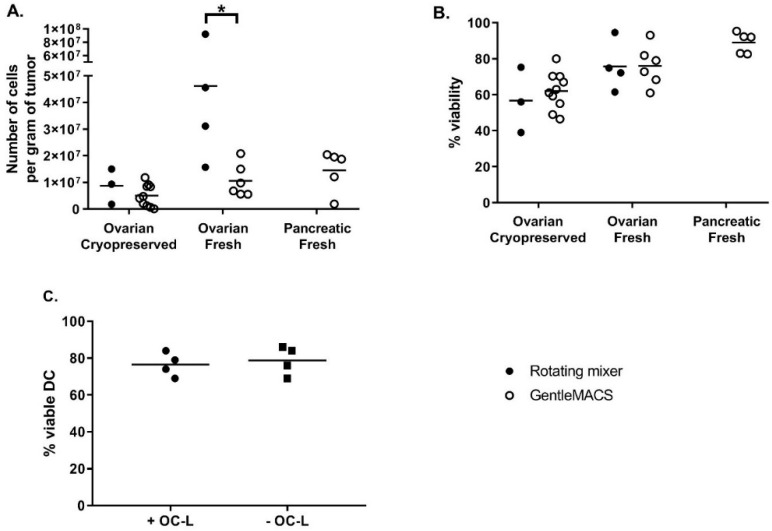
Oxidized tumor cell lysate (OC-L) tumor dissociation and impact of OC-L loading onto dendritic cell (DC). Cryopreserved or fresh tumor specimens were dissociated using an enzymatic digestion solution and incubated either on a rotating mixer at RT (closed symbols) or using the GentleMACS (open symbols). After dissociation, the total number of viable cells per gram of tumor (**A**) and percentage of viability (**B**) were determined. iDC were loaded or not with OC-L overnight, subsequently matured for 6 to 7 h using IFNγ and MPLA and viability of the cells was determined upon harvest (**C**, black circles with OC-L and black squares without OC-L). * Mann-Whitney test, *p* = 0.0041, n = 3 to 10.

The next phase consisted in the analysis and identification of steps where cell yield could be increased and/or manufacturing risk reduced. The tumor dissociation step was optimized by comparing the efficiency of tumor dissociation using either a rotating mixer (overnight at RT) or the GentleMACS dissociator (tumor dissociation program, 3 × 30 min at 37 °C with rotation). The efficiency was evaluated based on cell recovery per gram of tissue and cell viability. As shown in Figure 1A, the cell recovery from fresh samples was higher than that from cryopreserved samples, especially when using the rotating mixer method. The cell recovery per gram of tissue was also significantly higher when fresh ovarian tumors were dissociated using the rotating mixer method compared to fresh ovarian tumors dissociated using the GentleMACS (46.2 ± 33.1 × 10^6^ cells and 10.6 ± 6.2 × 10^6^ cells, respectively, Mann-Whitney test *p* = 0.019). As shown in Figure 1B, cell viability after dissociation was high and equivalent between both dissociation methods. Again, the viability appeared to be higher for fresh compared to cryopreserved tumors (75.8 ± 13.8% fresh vs. 56.8 ± 18.2% cryopreserved for ovarian tumors dissociated with rotating mixer; 76.1 ± 11.2% fresh vs. 62.2 ± 10.3% cryopreserved for ovarian tumors dissociated with GentleMACS and 89.1 ± 5.9% for fresh pancreatic tumors dissociated with GentleMACS). Our results demonstrate that this GMP-compliant tumor dissociation process allows for the isolation of a number of viable cells per gram of tissue sufficient to load an average of 92.4 × 10^6^ DC at a 0.5:1 OC-L: DC cell number ratio. Because of a higher efficiency of digestion using an overnight incubation at RT on a rotating mixer, we decided to use this method for OC-L clinical production.

Other than the change in the oxidative reagent, the oxidation and freeze-thaw cycle process was performed as described by Chiang et al. Importantly, after the last freeze-thaw cycle, the viability of the OC-L was controlled using Trypan blue exclusion staining. Over the 28 OC-L batches produced, 0% viability was always reached after six freeze-thaw cycles.

Nonetheless, one major risk to assess was whether the traces of HOCL remaining in the OC-L could impact the DC viability after loading. This was investigated by checking the viability of iDC loaded or not with OC-L after overnight (12 to 16 h) incubation and subsequently matured for 6 to 7 h using IFNγ and MPLA. As shown in Figure 1C, OC-L loading did not impact DC viability at harvest. Indeed, the viability of OC-L loaded DC (76.5 ± 6.5% viable cells) was comparable to viability of non-loaded DC (78.8 ± 7.8% viable cells).

Finally, from a quality control point of view, a colorimetric hypochlorite detection kit (Abcam) was used to detect the potential traces of HOCl in OC-L. Measurement demonstrated that HOCl level in the oxidized tumor lysate is below the limit of detection of the assay (i.e., 0.001%), thus confirming that this method is GMP compliant.

### 3.2. Validation of Monocytes Isolation Using the CliniMACS Prodigy

In order to perform monocytes isolation in a closed system compliant for GMP manufacturing in a Grade D clean room, we tested and validated the positive selection of monocytes from fresh leukapheresis using the CliniMACS CD14 reagent and the CliniMACS Prodigy system (Miltenyi Biotec).

Upon reception of the fresh leukapheresis material, the percentage of monocytes was defined by flow cytometry based on cell size and granularity (Forward scatter (FSC)/Side scatter (SSC)). Using this percentage, the CD14 positive selection was set-up on the CliniMACS Prodigy using the LP-14 enrichment program. After CliniMACS Prodigy priming and connection of the leukapheresis bag and the CliniMACS CD14 reagent to the tubing set, the selection procedure was automated and was completed within 2 to 4 h depending on the total number of cells and the percentage of monocytes present in the leukapheresis starting material. At the end of the enrichment, the target cell bag (CD14^+^ cells) and the non-target cell bag (CD14^−^ cells) were sealed off from the tubing set. CD14^+^ monocytes and CD14^−^ cells were counted and viability was determined by Trypan blue staining to determine the recovery percentage.

**Figure 2:**
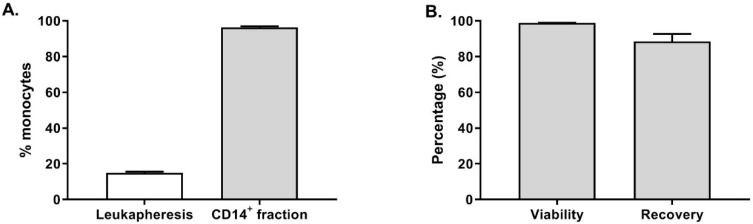
Monocytes purification by magnetic selection. (**A**) The percentage of monocytes (i.e., CD14^+^) was evaluated by flow cytometry on fresh leukapheresis (white bar, n = 21) and on the CD14^+^ fraction (grey bar, n = 17) after magnetic selection on the CliniMACS Prodigy. (**B**) Monocytes viability after purification (n = 20) as well as percentage of recovery from leukapheresis (grey bars, n = 21).

Furthermore, the fresh leukapheresis and the CD14^+^ fraction were stained to evaluate the purity of the monocytes. As shown in Figure 2A, the average percentage of CD14^+^ cells in the twenty-one fresh leukapheresis tested was 14.8 ± 3.5% (gated on live cells). After enrichment, 96.3 ± 2.8% of the cells were CD14^+^ cells in the target cell fraction, demonstrating an efficient CD14 enrichment resulting in highly pure monocytes. Furthermore, as shown in Figure 2B, the CD14^+^ cells viability was not affected by the enrichment procedure as 98.8 ± 0.9% of the cells were viable. Among the CD14^+^ cells detected in the leukapheresis before monocytes selection, 88.4 ± 19.7% were recovered in the target cell fraction at the end of the procedure.

These results clearly show the suitability and reliability of the CliniMACS Prodigy closed system to perform a reproducible and GMP-compliant CD14^+^ monocytes enrichment. On average, we collected of 1.3 ± 0.6 × 10^9^ CD14^+^ cells (n = 20 healthy donors) prior to starting moDC differentiation, which would be sufficient to seed 3–10 cell factories leading to the potential production of 150–500 × 10^6^ OC-DC or 15–50 vaccine doses.

### 3.3. Optimization of Monocytes Differentiation into Immature Monocyte Derived DC

Following the CD14^+^ monocytes enrichment, the culture process for the differentiation of monocytes into iDC was optimized (e.g., culture vessels) and validated by verifying whether the differentiation protocol described in [11,12] was applicable to monocytes positively selected using CD14 magnetic beads. To do so, CD14^+^ cells were enriched using the CliniMACS Prodigy and placed in culture for 5 to 6 days in DC medium (CellGenix DC medium supplemented with 2% human serum, IL-4 and GM-CSF). Several culture vessels were compared: cell factories (Thermofisher Scientific, Roskilde, Denmark), Vuelife Bags (Saint-Gobain, Courbevoie, France), and the CliniMACS Prodigy cell culture chamber. The latter was an obvious option as monocytes were enriched using the CliniMACS Prodigy. However, it should be noted that, to date, the capacity of the CliniMACS Prodigy cell chamber being limited to 260 mL, the cell density had to be increased from 1 × 10^6^ cell/mL to 3 × 10^6^ cell/mL in order to be able to culture sufficient cell numbers.

**Figure 3:**
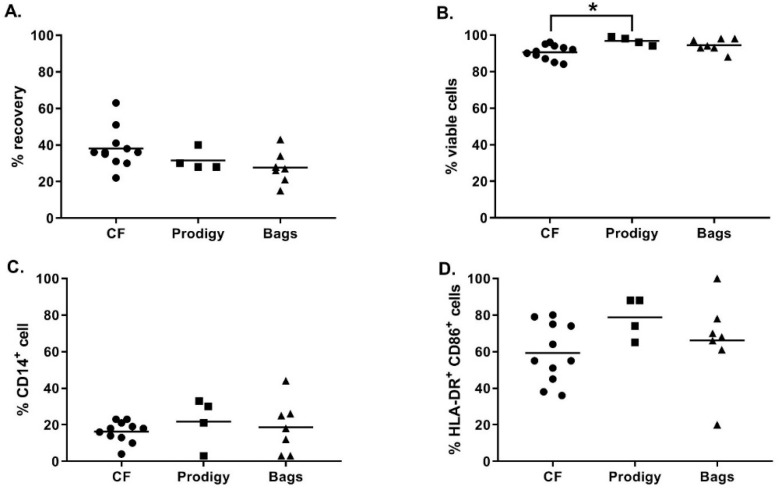
Characterization of immature DC (iDC) differentiated in cell factories, CliniMACS Prodigy, or Vuelife bags. After 5–6 days of culture, the percent recovery of iDC was calculated based on the number of monocytes put in culture at day 0 and the number of iDC at harvest for cells cultured in cell factories (CF, black circles), CliniMACS Prodigy (Prodigy, black squares) and Vuelife bags (black trianlgles) (**A**); the percentage of viable iDC at harvest (**B**); and phenotype analysis of the expression of CD14 (**C**); and HLA-DR/CD86 (**D**) by flow cytometry are also shown. * Kruskall–Wallis test, *p* = 0.0108, n = 4 to 11.

As shown in Figure 3A, at day 5, the recovery percentage of iDC was not significantly different between the culture vessels (38.1 ± 10.9% in cell factories, 31.5 ± 5.8% in the CliniMACS Prodigy cell culture chamber, and 27.7 ± 9.0% in Vuelife bags, Kruskal-Wallis test *p* ≥ 0.05). Moreover, no major difference was observed between the different culture vessels concerning the viability (Figure 3B) and phenotype of the iDC (Figure 3C–D). As expected, during monocyte to DC differentiation, we observed the upregulation of the percentage of HLA-DR^+^CD86^+^ cells and the downregulation of CD14 expression. The only significant difference observed was a higher viability of iDC cells cultured in the CliniMACS Prodigy culture chamber compared to iDC culture in cell factories (respectively 96.8 ± 2.2% and 90.6 ± 4.0%, Kruskal–Wallis, *p* = 0.0241). Altogether, these results show that, using the optimized differentiation protocol, magnetically enriched monocytes differentiated properly into highly viable iDC in all cell culture vessels tested.

### 3.4. Antigen Loading and Maturation of Monocyte-Derived DC (Day 5–6)

The next step was to evaluate the antigen loading and maturation of moDC steps. The process described by Chiang et al. [11,12] includes the use of cell factories and lipopolysaccharide (LPS) in combination with interferon-γ (IFNγ) as the maturation cocktail. As such, the use of the “open” cell factories and of LPS was evaluated.

Concerning the cell culture vessel, we wanted to implement the use of a closed system for cell culture by using the CliniMACS Prodigy culture chamber for antigen loading and maturation of DC. However, this did not appear as a suitable option as because of the dead volume to be taken into account for the transfer of reagents into the chamber, the antigen loading step ended up being highly lysate consuming. Furthermore, the addition of the maturation cocktail (LPS/IFNγ) led to the strong adherence of the DC to the CliniMACS Prodigy culture chamber and we were not able to detach the cells from the chamber.

**Figure 4:**
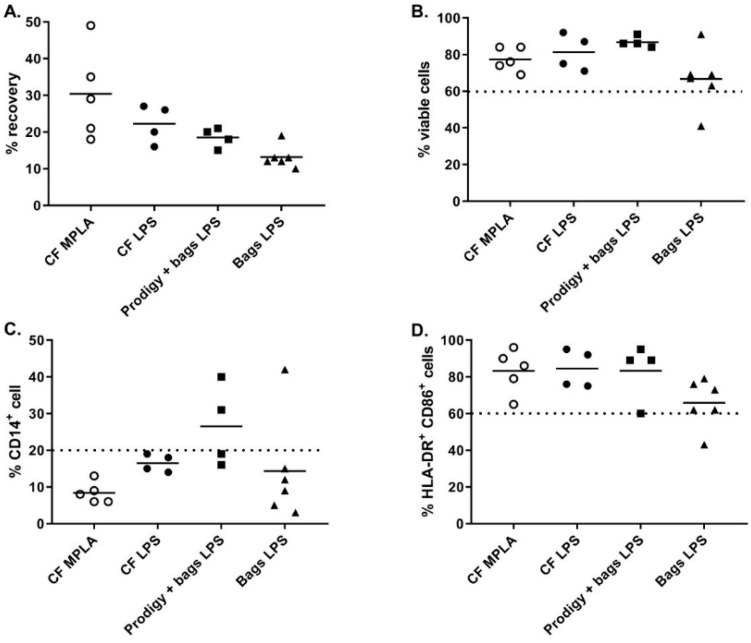
Characterization of mature DC differentiated in cell factories, CliniMACS Prodigy or bags. At day 6 (harvest) the differentiation and maturation of mDC was evaluated based on (**A**) percent recovery of mDC from number of monocytes put in culture, (**B**) percentage of viable mDC at harvest, and phenotype analysis of (**C**) CD14, and (**D**) HLA-DR/CD86 expression by flow cytometry. Open circles for CF MPLA, black circles for CF LPS, black squares for Prodigy/bags/LPS and black triangles for Bags/LPS. Statistics: Kruskall–Wallis test, n = 4 to 6.

An alternative closed system for cell culture was the Vuelife bags. The Vuelife bags were used either from day 0 to day 6 (Bags condition) or from day 5 to day 6 in combination with iDC differentiated in the CliniMACS Prodigy (Prodigy + bags condition). We compared both conditions to the culture in cell factories from day 0 to day 6 (CF conditions). As shown in Figure 4A, the recovery percentage of iDC tended to be higher when cells were cultured in cell factories compared to Vuelife bags or Prodigy + Vuelife bags culture conditions. For cells cultured in Vuelife bags from day 0 to 6, the cell viability was lower compared to other cell culture conditions (Figure 4B). Most importantly, the DC phenotype was negatively affected by the culture in the Vuelife bags as two out of four batches of DC produced in “Prodigy + bags” and one out of six batches of DC produced in “bags” were out of specification (≤20.0% CD14^+^ cells) for percentage of CD14^+^ cells (Figure 4C). Furthermore, the percentage of HLA-DR^+^ CD86^+^ cells in “bags” cultures (n = 6) was lower than in “CF” with one batch out of specification (≥60.0% HLA-DR^+^ CD86^+^ cells) and two batches at the lower limit of specification (Figure 4D). These results show that the cell factories performed better than the CliniMACS Prodigy and/or Vuelife bags with regards to the differentiation, antigen loading, and maturation of monocyte-derived DC.

**Figure 5:**
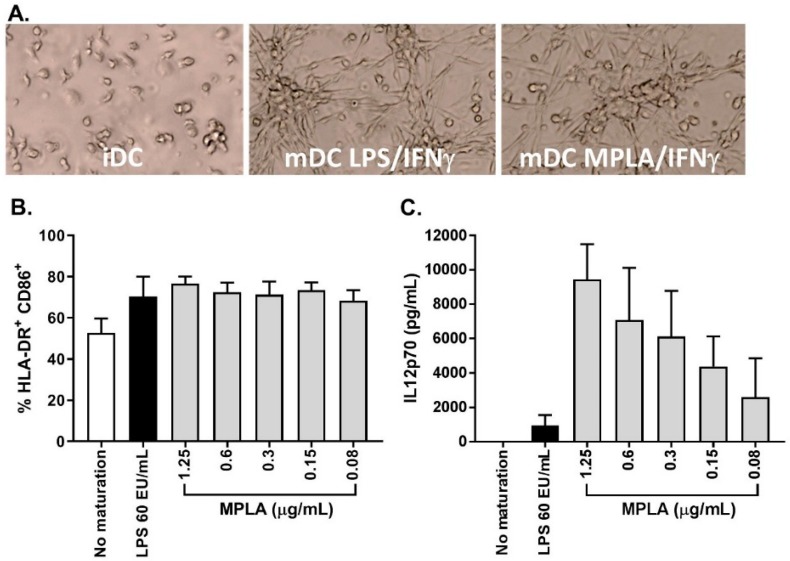
Maturation of iDC into mDC using LPS/IFNγ or MPLA/IFNγ. iDC were left immature (white bar) or matured overnight with either LPS (60 EU/mL) and IFNγ (2000 IU/mL) (black bar) or MPLA (0.6 µg/mL) and IFNγ (2000 IU/mL) (grey bars). Upon harvest, DC morphology (**A**), percentage of HLADR^+^CD86^+^ (**B**), and levels of IL12p70 in culture supernatant (**C**) were determined. Statistics: Kruskall–Wallis test, n = 2 to 3.

As stated previously, one major change from the previously published process was the substitution of LPS to monophosphoryl lipid A (MPLA) as a maturation agent. Indeed, the high toxicity level of LPS excludes its use for clinical applications, whereas MPLA represent a low toxicity derivative of LPS available in GMP grade. In combination with IFNγ, MPLA was shown to be an efficient maturation agent for monocyte-derived DC maturation [14,15,16]. As such, we integrated the use of MPLA in the manufacturing process, first by titration using small-scale experiments (24-well culture plates) and by comparing its efficiency to LPS (Figure 5A). The percentage of CD14^+^ cells in mDC was ≤20.0% for all maturation cocktails tested and the percentage of HLA-DR^+^/CD86^+^ cells was comparable between LPS-IFNγ and MPLA-IFNγ suggesting that MPLA was a good alternative to LPS for DC maturation (Figure 5B). Interestingly, secretion of IL12p70 was higher when cells were matured with MPLA/IFNγ (Figure 5C). As the viability tended to drop when 1.25 µg/mL MPLA was used, we decided to use 0.6 µg/mL MPLA in combination with 2000 IU/mL IFNγ as a maturation cocktail.

MPLA-IFNγ and LPS-IFNγ maturation cocktails were finally compared in full-scale conditions using cell factory cultures. As shown in Figure 4, while the cell viability and percentage HLA-DR^+^/CD86^+^ cells were comparable between the two maturation cocktails, we observed a higher recovery and a lower CD14^+^ cells percentage when using MPLA-IFNγ compared to LPS-IFNγ as a maturation cocktail.

Altogether, these results show that the MPLA-IFNγ maturation cocktail is a good alternative to the LPS-IFNγ, as well as being safer.

### 3.5. OC-DC Cryopreservation and Functional Testing

**Figure 6:**
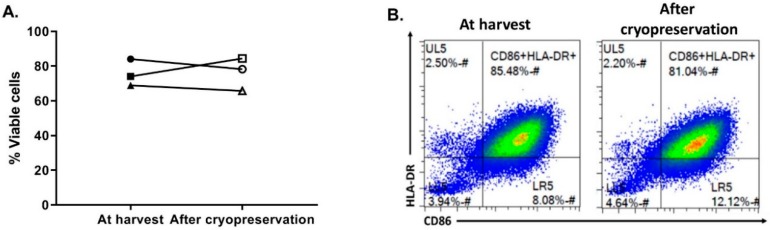
OC-DC cryopreservation. At day 6 after 6–8 h of maturation with MPLA/IFNγ, OC-DC cells were harvested and analyzed either fresh (black symbols) or after cryopreservation in CS10 (open symbols). OC-DC viability (**A**) and phenotype (**B**) at harvest and after cryopreservation were analyzed. The different symbols shapes represent different batches. Statistics: paired *t*-test.

The last critical process step to evaluate was the OC-DC cryopreservation. First, in order to limit the use of human serum, the CS10 (Biolife Solutions) was selected as the cryopreservation solution. To confirm the suitability of using CS10, OC-DC cells were harvested, washed in DPBS, and cryopreserved at 5–10 × 10^6^ cell/mL vaccine doses. After storage in liquid nitrogen, OC-DC doses were thawed with the viability and phenotype evaluated. As shown if Figure 6, compared to OC-DC cells prior to cryopreservation, the cryopreservation of OC-DC doses in CS10 did not impact the viability (75.6 ± 7.7% vs. 76.1 ± 9.5% at freezing vs. thawing, respectively, Figure 6A) nor the phenotype (Figure 6B). Moreover, in our hands the percentage of CD14^+^ cells was not affected by cryopreservation.

Next, the functionality of the OC-DC doses was tested using both the COSTIM assay [17,18] and by measuring the level of IL12p70 in culture supernatant after at least 6 h of maturation. The COSTIM assay demonstrated that OC-DC cells were able to induce CD4 and CD8 T cell proliferation in the presence of a suboptimal amount of anti-CD3 in a 3-day co-culture of autologous OC-DC and T cells (Supplementary Figure S1). Interestingly, when we compared the functionality of OC-DC cells cryopreserved in 10% DMSO/90% human serum or CS10, it appeared that cells cryopreserved in CS10 induced higher T-cell proliferation, especially for CD8 T cells with 84.8 vs. 64.0% proliferating CD8 T cells for CS10 vs. 10% DMSO/90% human serum, respectively. The level of IL12p70 in culture supernatant after maturation was measured by ELISA and showed that all OC-DC cells tested produced sufficient amount of IL12p70 and met the specification of ≥50 pg/mL with a high variability between batches (from 70 to 4126 pg/mL). Taken together, these results suggest that the OC-DC vaccine doses produced were functional with the OC-DC phenotype stable upon cryopreservation in CS10.

### 3.6. Process Validation Using Full-Scale OC-DC Manufacturing

**Figure 7:**
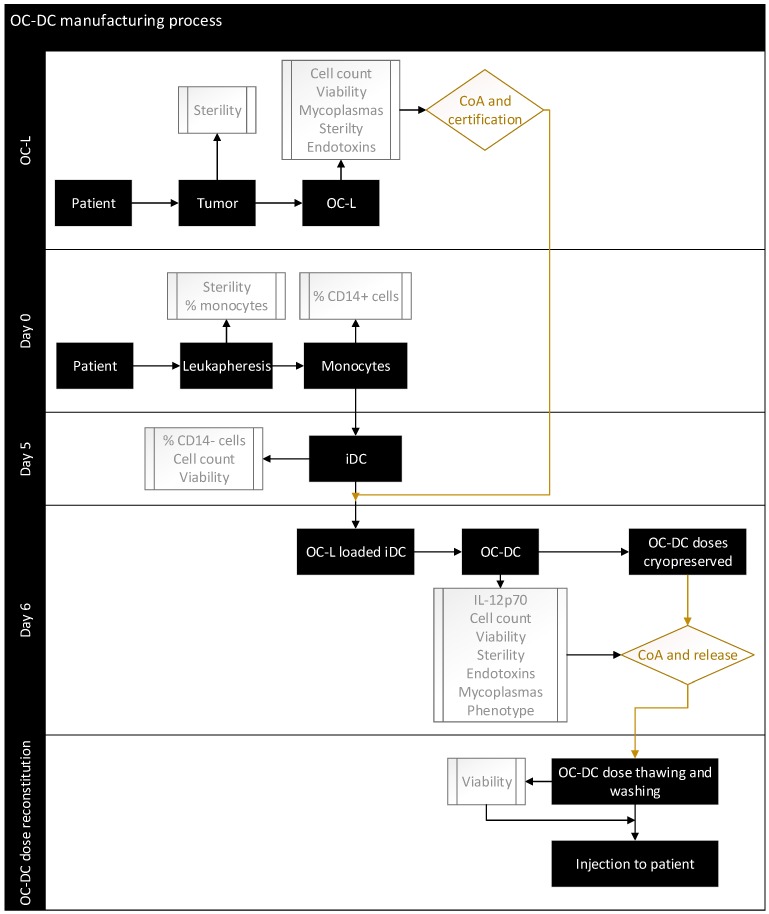
OC-DC manufacturing flow chart. Flow chart of the OC-DC manufacturing process with process steps shown as black boxes, in process and quality controls shown as grey boxes, and certification/release steps shown as diamond-shaped boxes.

Having set the culture conditions using a GMP-compliant process, we performed full-scale OC-DC manufacturing in a grade D clean room environment, under a Grade A isolator for open steps, based on the flow diagram shown in Figure 7.

Full-scale manufacturing included the quality control strategy suitable for phase I clinical trials. The classification of starting materials, intermediate products, active substances, and final product was defined in accordance with the local authorities (Swissmedic) as described in Table 1.

OC-DC vaccine production was performed using three (3) healthy donors leukapheresis and OC-L produced from ovarian tumors as a source of antigens. The monocytes were differentiated into iDC in cell factories with one satellite cell factory used at day 5 to evaluate the number of iDC recovered. Based on the number of iDC from this satellite flask, the OC-L concentration was determined and the iDC were loaded with OC-L at a 0.5:1 ratio (OC-L:iDC cell number) overnight and matured on day 6 with the addition of MPLA-IFNγ for 6 to 8 h before harvest.

The quality control strategies of the two active substances (OC-L and iDC), including the specifications of in-process controls and final product release testing are described in Table 2 and Table 3.

**Figure 8:**
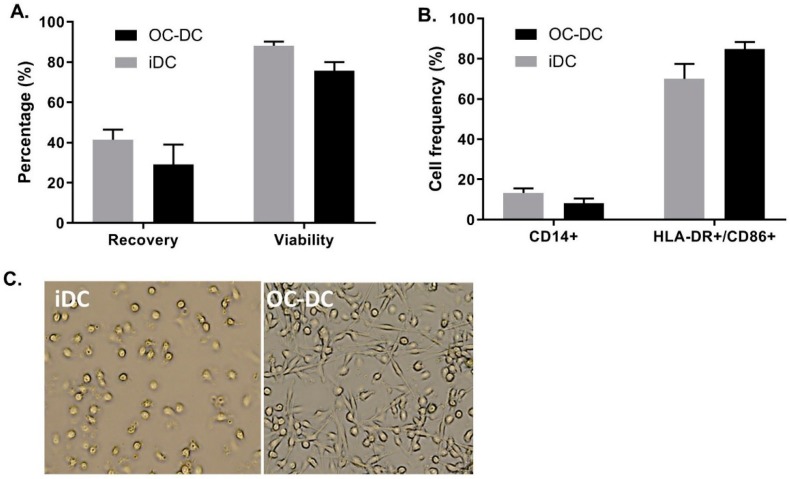
Large scale OC-DC batches: cellular characterization. After 5 days of culture, OC-L was added at a 0.5:1 (OC-L cells:DC) ratio overnight. The day after, DCs were matured for 6–8 h with MPLA and IFNγ. The percent recovery of iDC and OC-DC (**A**) the percentage of viable iDC and OC-DC, the phenotype analysis (**B**) and the DC morphology (**C**) before (iDC) and after (OC-DC) 6 h of maturation were evaluated.

As shown in Figure 8A, we recovered 41.4 ± 8.7% of iDC and 29.0 ± 17.4% of OC-DC from CD14^+^ cells placed in culture at day 0. This extrapolates to a potential production of 8.0% ± 1.2 × 10^10^ iDC resulting in 5.2% ± 1.6 × 10^8^ OC-DC, which would correspond to 103.5 ± 31.1 doses of 5 × 10^6^ OC-DC. Nonetheless, the limiting step in the number of potential vaccine doses produced remain the amount of OC-L available for antigen loading. Additionally, viability remained constantly high for both iDC and OC-DC at harvest (respectively 88.2 ± 3.4% and 75.6 ± 7.7% viable cells), (Figure 8A) suggesting the high quality of the OC-L. Small-scale experiments were representative of the three large-scale batches of OC-DC performed since the DC phenotype of the large scale batches also met the phenotype specification (≤20.0% CD14^+^ cells and ≥60.0% HLA-DR^+^ CD86^+^ cells), (Figure 8B) with DC maturation leading to a decrease in CD14^+^ cells and an increase of HLA-DR^+^/CD86^+^ cells (Figure 8B). The morphology of the cells before and after maturation confirmed previous data, with loosely adherent round iDC and highly adherent elongated OC-DC (Figure 8C).

**Figure 9:**
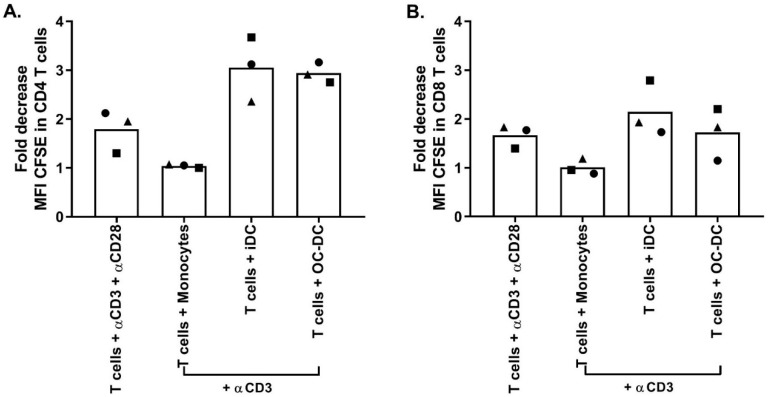
Evaluation of OC-DC using COSTIM assay. Autologous T cells were purified from PBMC, labelled with CFSE and co-cultured with thawed OC-DC cells. After 3 days of culture, cells were harvested and analyzed by flow cytometry. Results are presented as fold decrease in CFSE MFI compared to respective controls of CD3-CD28, monocytes or iDC in viable CD4 and CD8 T cells in panels (**A**,**B**) respectively. Monocytes were used as a negative control and stimulation with αCD3 and αCD28 was used as a positive control. The different symbols shapes represent different batches of cells. Statistics: Kruskall–Wallis test, n = 3.

Similar to small-scale experiments, The functionality was again evaluated by COSTIM assay and by IL12p70 ELISA. The COSTIM assay showed that OC-DC cells induced the proliferation of both CD4+ and CD8+ T cells with a decrease in the CFSE MFI observed in the presence of anti-CD3 in the culture compared to OC-DC only (Figure 9). As a negative control, autologous CD14^+^ cells did not induce T-cells proliferation (no decrease in CFSE MFI). As a positive control, T cells stimulated with both anti-CD3 and anti-CD28 induced T-cell proliferation. The OC-DC functionality observed in the COSTIM assay was confirmed by the presence of IL12p70 in the culture supernatant of OC-DC at levels above specification (≥50 pg/mL). As the measurement of the IL12p70 level in culture supernatant is a fast and reliable readout for DC functionality, we included it as part of the final release testing for OC-DC batch certification.

Finally, the impact of the reconstitution process on product stability was tested and optimized based on the formulated product phenotype and viability over time to evaluate. The OC-DC dose specification is described in Table 4.

Briefly, OC-DC doses’ reconstitution included a thawing step using a thawing solution, a washing step by centrifugation, a step where the resuspension of the OC-DC dose in 0.9% NaCl 1% Albumin occurred, and finally a step where the transfer of the OC-DC dose in two 1-mL syringes to allow for injection (sub-cutaneaous or intra-nodal) at two sites. The OC–DC doses were kept at 2–8 °C after reconstitution as DC are known to be temperature sensitive and would adhere if left at RT or 37 °C. The syringes are labelled and packed according to local procedures and kept at 2–8 °C until injection.

**Figure 10:**
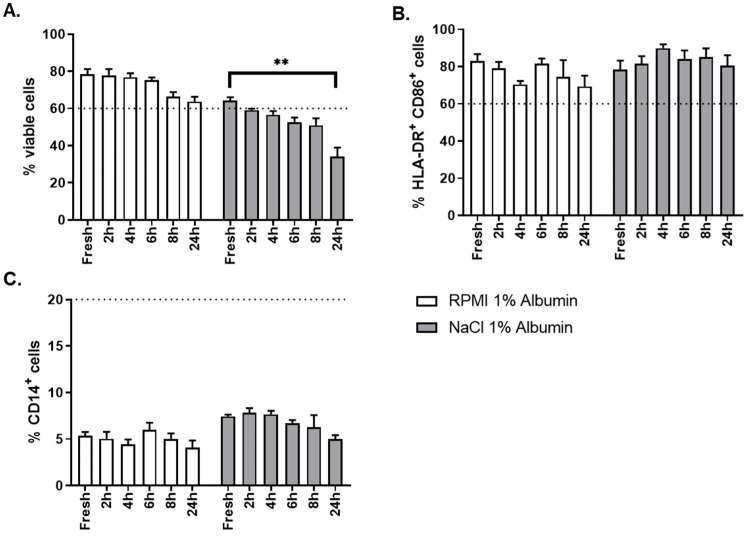
Stability of OC-DC following formulation. Stability of OC-DC formulated doses was evaluated for three OC-DC batches. Two thawing solutions were compared: RPMI 1% albumin (white bars) and 0.9% NaCl 1% albumin (grey bars). Stability of the formulated OC-DC doses was evaluated based on the percentage of viable cells after formulation (**A**) and phenotype analysis of HLA-DR/CD86 (**B**) and CD14 (**C**) expression by flow cytometry. Data points represent the mean value of 1 to 3 repeats per batch per time point. ** Kruskal–Wallis, *p* ≤ 0.01, n = 3.

The critical aspect evaluated for this step was the choice of the thawing solution. Two different thawing solutions were evaluated: NaCl 0.9% albumin 1% and RPMI albumin 1%. As shown in Figure 10A, thawing of OC-DC in RMPI 1% albumin gave better results than NaCl 0.9% albumin 1% in terms of cells viability, not only at time of thawing (fresh sample), but also over time (up to 24 h after formulation) after thawing. OC-DC cells met viability specification (≥60.0% viable cells) until 8 h after formulation when RPMI albumin 1% was used, whereas two batches were already out of specification at 2 h after formulation with NaCl 0.9% albumin 1%. The phenotype was not influenced by the type of thawing solution used (Figure 10C).

Taken together these results demonstrate that RPMI 1% albumin is the optimal thawing solution for the reconstitution of OC-DC doses.

Based on these results, the OC-DC dose reconstitution process is composed of the following steps:

1. OC-DC dose thawing in RPMI 1% Albumin.
2. Washing by centrifugation and removal of the supernatant.
3. Resuspension of the OC-DC dose in 0.9% NaCl 1% albumin.
4. Transfer of the OC-DC dose into two 1-mL syringes to allow for injection (sub-cutaneaous or intra-nodal) at two sites.
5. OC-DC syringes labelling and packing according to local procedures and storage/transport at 2–8 °C until injection.

The OC-DC dose reconstitution is performed at the GMP manufacturing site (Grade D clean room under a Grade A isolator) before the transport of the reconstituted doses to the clinic ready for injection into the patient.

## 4. Discussion

The interest for DC-based vaccines was recently reinvigorated by their potential use in combination with other immunotherapies, such as immune checkpoint inhibitors and adoptive T-cell therapies. However, challenges remain regarding the manufacturing process time, cost effectiveness and GMP-compliance. Here, we present an optimized personalized DC vaccine production process using tumor lysate as tumor antigen source and monocyte-derived DCs as APCs in a closed system manufacturing process.

The tumor lysate preparation was optimized to produce sufficient amount of material with the required quality attributes. The OC-L process described by Chiang et al. [11,12] was adapted to the use of GMP-compliant reagents (buffers, enzymes, HOCl). To our knowledge, it is the first time a fully GMP-compliant oxidized tumor lysate production process is described. Because automated dissociation could reduce process time, we compared a passive dissociation method (digestion on a rotating wheel overnight at RT) and an active automated dissociation method (1.5 h digestion using the GentleMACS dissociator). In our hands, passive dissociation generated superior yields in terms of cell number recovered per gram of tumor. The mechanical stress caused by the GentleMACS tube helix may result in loss of cells even if other studies showed similar [19] or improved [20] yields using the GentleMACS dissociator. Nonetheless, the processing methods used in these studies were not comparable to the digestion on a rotating wheel overnight at RT presented here. Indeed, we describe a passive dissociation using digestion enzymes without mechanical stress, while Eyrich et al. [19] and Nava et al. [20] performed a mechanical dissociation using syringes. This reference method might be as harsh as the GentleMACS for the tissue, and hence the comparable results. Furthermore, our readout was the number of live cells after dissociation while other groups report quantity of proteins in the final tumor lysate.

A major safety attribute regarding the use of tumor lysate is the lack of viable cells at the end of the process, as well as conformity of the validated quality control strategy. Our process proved to be highly efficient in that regard as all OC-L batches generated (n = 28) contained no viable cell at the end of the process (dissociation, oxidation, and 5 freeze/thaw cycles). Quality controls performed to detect the presence of endotoxin (Endosafe, release criteria: ≤5 EU/mL) and mycoplasma (MycoSEQ, release criteria: negative), as well as sterility testing (BacTEC, release criteria: no growth) passed for the three batches tested. Additionally, we could not detect any trace of HOCl in the OC-L final product, and observed no detrimental effect of the use of HOCl for OC-L production on DC upon lysate loading. Consequently, this OC-L process was considered optimized for GMP-compliant manufacturing and was approved as such by Swissmedic for a phase I clinical trial.

Next, the CD14+ monocyte enrichment was optimized using the CliniMACS Prodigy system. This automated device allows for cell processing with minimal operator intervention using a GMP-compliant closed single-use tubing set. Once the leukapheresis starting material is ready, the processing buffer and the CD14 reagent are connected to the tubing set and that the enrichment parameters are set (i.e., cell concentration, volumes), the CliniMACS Prodigy performs the cell fractionation, the washing, the labeling and the separation automatically [21]. As shown by our results, this allows for a highly reproducible monocyte enrichment with high recovery and viability of the purified cells. This is in contrast with the results obtained using elutriation for monocytes enrichment [22,23] for which a higher variability in monocytes purity was observed. Even though the elutriation is a closed process, a low monocyte purity could lead to additional manufacturing steps to further enrich in monocytes. In a grade D clean room, these additional manufacturing steps (including cell factory handling) would represent a risk as any additional processing steps would increase the risks associated with open steps to be performed in a Grade A isolator in the DC vaccine production process. A drawback of the use of a tubing set with numerous luer-locks is that precautions should be taken to ensure the integrity of the tubing set (all luer-locks are properly closed) before starting the selection process. Furthermore, the cost of the CliniMACS Prodigy tubing sets and reagents is high and should be taken into account when estimating the benefit of gain of time and operator intervention vs. the cost of the process by itself.

As we previously [11] used elutriation to enrich monocytes, the potential impact of a positive selection using magnetic beads on monocytes differentiation into DC had to be evaluated. Results show that CD14 positive selection does not impair subsequent differentiation of monocytes into functional DC. It rather gives even higher yields than reported by other groups using an elutriation method [24,25] as 30.4 ± 12.4% of CD14^+^ cells seeded at day 0 in cell factories were recovered on day 6 after lysate loading and maturation using MPLA and IFNγ. For clinical trial application, the magnetic beads used for monocytes selection (CliniMACS CD14 reagent) should be considered as impurities in the final product.

The use of a closed system for cell culture is highly advantageous from a GMP standpoint as it considerably reduces the risk of sterility loss and potential contamination. Furthermore, in clean rooms with a Grade D environment where open steps are required to be performed under a Grade A isolator, using closed systems and sterile connections facilitates greatly the operator manipulations while reducing the workload under the isolator. Having this in mind, we tried to generate moDC in different culture vessels: cell factories, bags, or the culture chamber of the CliniMACS Prodigy. Regarding the culture in bags, as already described by others [25], DC derived from monocytes in such conditions had a decreased viability and a less mature phenotype than cells cultured in cell factories. Additionally, moDC differentiated and matured in bags secreted very low levels of IL12p70 compared to cultures in cell factories. These results led to the conclusion that bags were not suitable for moDC differentiation and maturation as they impaired cells phenotype and functionality.

Concerning the culture of cells in the CliniMACS Prodigy, two (2) critical drawbacks were identified. First, because of the restricted volume of the culture chamber (260 mL), and in order to generate enough cells to produce a batch of DC at the clinical size, the cell density was increased from 1 x 10^6^ monocytes/mL to 3 × 10^6^ monocytes/mL. Even if the results on differentiation of monocytes into iDC at such a high concentration did not show any impaired iDC phenotype, phenotype and/or functionality testing of mature mDC was not possible as the maturation in the culture chamber using LPS/IFNγ resulted in strong adherence of cells to the plastic and the mDC could not be retrieved. Second, the addition of OC-L to the culture chamber also proved to be challenging and required a larger amount of OC-L than for culture in cell factories because of the dead volume inherent to the tubing length. Approximately 10% more OC-L is needed for loading in the culture chamber compared to the loading in cell factories, and as the OC-L is the limiting factor in the number of OC-DC vaccine doses produced, this was considered a critical factor. Altogether, these results show that the differentiation, OC-L loading, and maturation of moDC in the CliniMACS Prodigy were not suitable for our process. Furthermore, if the cell culture was done in the CliniMACS Prodigy chamber, one device would be dedicated to the production of one OC-DC batch for the entire manufacturing process (6 days). This would strongly reduce the number of batches that could be produced weekly using this device.

An alternative option would be to differentiate monocytes into iDC in the Prodigy (day 0 to day 5), harvest iDC and transfer in bags for OC-L loading and maturation (day 5 and 6). Unfortunately, with this approach, the operator hands-on time significantly increases on day 5 and 6 compared with cultures in cell factories from day 0 to 6 for which no cell transfer is necessary. Additionally, the phenotype of the cells cultured in “Prodigy + bags” was impaired (increased CD14 percentage) when compared to cells cultured in cell factories alone, especially regarding CD14 expression, which suggest an impaired differentiation of the cells using “Prodigy + bags.” As such, even if the cell factories are not considered to be a closed system, they proved to be the most suited culture vessel for differentiation, OC-L loading, and maturation of OC-DC. Tubing connection are currently being tested on the cell factories therefore allowing their use as a closed system flasks inside a Grade D clean room.

Finally, the use of MPLA instead of LPS in the maturation cocktail needed to be optimized. It has already been shown that the use of MPLA/IFNγ to mature moDC was efficient to induce functional cells [14,15,16] and results presented here confirmed this observation. The use of MPLA/IFNγ led to higher secretion of IL12p70 by mature moDC than with LPS/IFNγ. The phenotype was as expected for mature moDC (≥60.0% HLA-DR^+^ CD86^+^ cells, ≤20% CD14^+^ cells) and similar with both maturation cocktails. Because of its non-toxicity and its availability as a GMP-grade reagent, MPLA was chosen in the maturation cocktail.

As the induction of antigen specific cytotoxic T lymphocytes by the OC-DC vaccine is difficult to study because of the unknown composition of the OC-L, the functionality of OC-DC was tested using two methods: the COSTIM assay and the detection of IL12p70 in culture supernatant after maturation by ELISA. Of note, we previously demonstrated the potential of OC-DC vaccines to induce T-cell response to autologous tumor antigen [11,13,26]. The COSTIM assay was adapted from Shankar et al. [17,18] and allows for T-cell proliferation to be evaluated by flow cytometry rather than by tritiated thymidine incorporation. This assay measures co-stimulatory activity, but not antigen processing and presentation by the DC. This method gave clear evidence that OC-DC produced using our process can induce a T-cell response. These results are in line with the IL12p70 secretion, with the cells secreting IL12p70 being also able to induce a T-cell response. For a matter of logistic and efficiency, we decided to include only IL12p70 measure as a potency assay for the release of the OC-DC vaccine doses (≥50 pg/mL IL12p70 in culture supernatant 6–8 h after maturation). Importantly, all quality controls performed on the final product (OC-DC at harvest) to detect the presence of endotoxin (Endosafe, release criteria: ≤10 EU/mL) and mycoplasma (MycoSEQ, release criteria: negative), as well as sterility testing (BacTEC, release criteria: no growth) passed for the three batches tested confirming safety of the OC-DC product.

Finally, full-scale batches confirmed the feasibility of our manufacturing process with the potential generation of 103.5 ± 31.1 doses of 5 × 10^6^ OC-DC, prior to considering the availability of OC-L. Indeed, as the mDC differentiation proved to be very efficient in terms of cell number produced, the limiting factors identified were (1) the OC-L availability and (2) the quantity of circulating monocytes present in cancer patients. For the latter, this emphasizes the importance of the selection of patients to be included in clinical trials based on the use of OC-DC. Of course, an indication of choice for the use of OC-DC is ovarian cancer since it has already been shown that treatment of advanced epithelial ovarian cancer patients with OC-DC in combination with bevacizumab and cyclophosphamide is feasible and safe [13]. However, all patients presenting solid tumors big enough (several grams) to allow for the production of a sufficient amount of OC-L (at least 45 × 10^6^ cells) would be suitable for OC-DC vaccines.

## 5. Conclusions

In this report, we presented a manufacturing process for OC-DC vaccines based on the use of oxidized tumor lysate and monocyte derived dendritic cells. The feasibility of using a fully GMP-compliant process was demonstrated for a use in a Grade D clean room environment using a Grade A isolator for open steps. This reproducible and standardized OC-DC production process could be used as a reference for DC vaccines production. Indeed, the use of the CliniMACS Prodigy and its automated features for monocytes enrichment should facilitate technical transfer between manufacturing centers independently of the classification of their clean rooms (Class D to A). Importantly, we are the first group to describe the production of a DC-based vaccine using the CliniMACS Prodigy in combination with culture in cell factories and an MPLA and IFNγ-based maturation. The OC-DC production process was approved by Swissmedic and we conducted two clinical trials utilizing the two types of personalized DC vaccines in two different indications (ovarian and pancreatic cancer) [27,28].

## Figures and Tables

**Table 1:** Definitions of the OC-DC starting materials, intermediate products, active substances, and final product.

Name	Definition
**Starting materials**	OC-DC vaccines are composed of two starting materials: The patient tumor-derived biopsy obtained following surgery that serves to produce oxidized tumor cell lysate (OC-L); The patient leukapheresis from which monocytes are purified and used to produce monocyte-derived DC.
**Intermediate product**	For the OC-DC process the intermediate product is: Cryopreserved dissociated tumor cells (if applicable).
**Active substances**	OC-DC vaccines are composed of two (2) active substances: Oxidized tumor cell lysate (OC-L); Autologous immature monocyte-derived DC.
**Final product**	The final product represents the cryopreserved OC-DC doses (autologous mature monocyte-derived dendritic cells (DC) pulsed OC-L).
**Final reconstituted dose**	Individual OC-DC doses formulated 0.9% NaCl 1.0% Albumin.

**Table 2:** Active substances (OC-L and iDC) specifications.

Active Substance	Test	Analytical Procedure	Specification
OC-L	Cell viability	Trypan blue exclusion	0.0% viable cells
	Sterility	BacTEC (aerobic, anaerobic)	No growth
	Mycoplasma	MycoSeq	Negative
	Endotoxin	Endosafe	≤5.0 EU/mL
iDC	Viability	Trypan blue exclusion	≥80.0% viable cells
	Cell count	Neubauer hemacytometer	Percent recovery (no specification)
	Phenotype	Flow cytometry	≥70.0% CD14 negative cells

**Table 3:** Final product (OC-DC) specifications.

Specimen	Test	Analytical Procedure	Specification
OC-DC	Sterility	BacTEC (aerobic and anaerobic)	No growth
	Mycoplasmas	MycoSeq	Negative
	Endotoxins	Endosafe	≤10.0 EU/mL
	Cell count	Manual cell count by Trypan blue exclusion	≥45.0 × 10^6^ viable cells
	Viability		≥60.0% viability
	Phenotype	Flow cytometry	≥60.0% live HLA-DR^+^CD86^+^ cells ≤20.0% CD14^+^ cells
OC-DC culture supernatant	IL-12p70	ELISA	≥50.0 pg/mL

**Table 4:** Reconstituted OC-DC dose specification.

Specimen	Test	Analytical Procedure	Specification
Reconstituted final dose	Viability	Trypan blue exclusion	Target: ≥60.0% viability

## Supplementary Materials

The following are available online at https://www.mdpi.com/2076-393X/8/1/25/s1. Figure S1: Evaluation of OC-DC cells functionality after cryopreservation using COSTIM assay.
