# Supplementary material for: Development and Optimization of a GMP-Compliant Manufacturing Process for a Personalized Tumor Lysate Dendritic Cell Vaccine
Source: Vaccines (Basel). 2020 Jan 14;8(1):25. doi: 10.3390/vaccines8010025 (PMC7157441; doi:10.3390/vaccines8010025)
Supplement: Supplementary file 1 [file vaccines-08-00025-s001.pdf]

vaccines

Article

Development and Optimization of a GMP-Compliant Manufacturing Process for a Personalized Tumor Lysate Dendritic Cell Vaccine

Caroline Boudousquié [1,*], Valérie Boand [1], Emilie Lingre [1], Laeticia Dutoit [1], Klara Balint [1], Maxime Danilo [2], Alexandre Harari [1,2], Philippe O. Gannon [1] and Lana E. Kandalaft [1,2,*]

[1] Department of Oncology, Centre Hospitalier Universitaire Vaudois, Lausanne Postal Code, Switzerland; valerie.boand@gmail.com (V.B.); Emilie.Lingre@chuv.ch (E.L.); Laeticia.Dutoit@chuv.ch (L.D.); Klara.Balint@chuv.ch (K.B.); Alexandre.Harari@chuv.ch (A.H.); Philippe.Gannon@chuv.ch (P.O.G.);

[2] Department of Oncology, Ludwig Institute for Cancer Research, University of Lausanne, Lausanne Postal Code, Switzerland

* Correspondence: Caroline.Boudousquie@chuv.ch (C.B.); Lana.Kandalaft@chuv.ch (L.E.K.)

Received: 11 December 2019; Accepted: 27 December 2019; Published:

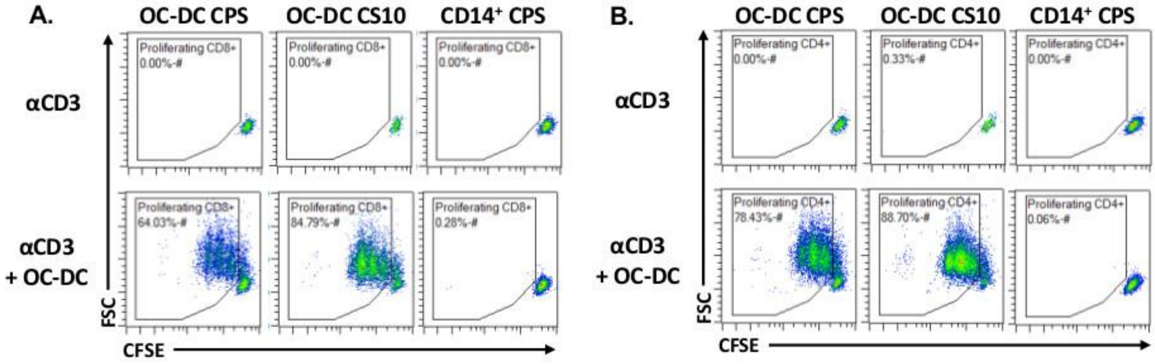

Figure S1. Evaluation of OC-DC cells functionality after cryopreservation using COSTIM assay. OC-DC cells were harvested at day 6 after maturation and cryopreserved in two (2) different cryomedia: CPS (90% HS 10% DMSO) or CS10. Autologous T cells were purified from PBMC, labelled with CFSE and co-cultured with OC-DC cells. After 3 days of co-culture, cells were harvested and analysed by flow cytometry. A decrease in CFSE mean fluorescence intensity compared to negative control (T cells +anti-CD3) identifies proliferating cells. Results are presented as the percentage of **(A)** CD4 and **(B)** CD8 viable proliferating T cells. CD14+ monocytes cryopreserved in CPS were used as a negative control.

© 2020 by the authors. Submitted for possible open access publication under the terms and conditions of the Creative Commons Attribution (CC BY) license (http://creativecommons.org/licenses/by/4.0/).
